# Implants in the Anterior Maxilla: Aesthetic Challenges

**DOI:** 10.1155/2015/152420

**Published:** 2015-06-11

**Authors:** Sang-Choon Cho, Stuart J. Froum, Angela R. Kamer, Peter M. Loomer, Georgios Romanos, Burak Demiralp

**Affiliations:** ^1^Periodontology and Implant Department, New York University, College of Dentistry, New York, NY 10010, USA; ^2^Stony Brook University, School of Dental Medicine, Stony Brook, NY 11790, USA; ^3^Hacettepe University, Faculty of Dentistry, 06100 Ankara, Turkey

Esthetic outcomes have become key elements that are critical to defining success in implant restorations. Long-term studies have demonstrated that single or multiple implants are highly predictable with high survival rates. However, in the anterior maxillary zone, the aesthetic success of implant therapy is, for many, as important as the implant survival rates. Several factors contribute to this “success” and can be objectively evaluated. These include the patient's healing capabilities, the level and condition of the existing soft and hard tissues, and the provisional and final restorations. In addition to these objective factors, esthetic perception also plays a significant role in achieving this “success.” This special issue contributes to the growing body of existing literature by examining several important issues related to the aesthetic aspects of maxillary implants and their increasingly important role in implant dentistry. These include esthetic perception, surgical techniques for tissue augmentation, and the esthetics of final implant-supported restorations.

One of the esthetic determinants occurring after implant placement is the lack of papilla between implants or between teeth and implants. Whether or not the lack of the interdental papilla leads to significant cosmetic deformities depends on whether shorter papillae may be esthetically tolerated under certain circumstances. Y. C. P. Yu et al. investigated the difference in the perception of aesthetics according to dental specialty. To this goal, the authors used computer assisted asymmetric alteration of the papilla length in the esthetic zone. The results of this study showed that asymmetric deficiencies in papilla length of 2 mm or more are more likely to be perceived as “unattractive.” It should be underlined that many dental professionals perceive even minor asymmetric shortening of the papilla as unattractive.

The stability and health of the peri-implant soft tissues are necessary for success and long-term maintenance of dental implants. A two-millimeter wide band of keratinized tissue has long been considered clinically desirable to provide a soft tissue seal around natural teeth. The absence of periodontal ligament and supracrestal fiber attachment around dental implants may increase peri-implant tissue susceptibility to the inflammatory process caused by biofilm accumulation. Therefore, creation of a band of keratinized gingiva around implants is desirable. A. Elkhaweldi et al. in a case series presented a novel surgical technique that can be utilized to augment keratinized soft tissue around implant-supported overdentures. The authors concluded that an apically repositioned flap, a relatively simple procedure, provided good esthetic outcomes, with newly formed tissue indistinguishable from the surrounding mucosa. The disadvantages of this technique are also discussed.

Full arch implant-supported prostheses have high success rates. A variety of materials have been used for this type of restoration including metal alloy-acrylic, metal alloy-composite, and metal alloy-ceramic. However, with these materials a number of complications may occur. To minimize these, using zirconia for the framework has been proposed. Several studies have shown that zirconia possesses excellent physical, mechanical, biological, and chemical properties. J. Carames et al. discuss this option and provide examples of monolithic zirconia restorations for full arch implant-supported restorations.

A. E. Borgonovo et al. investigated the survival and success rates, as well as the marginal bone loss (MBL) and esthetic indices of zirconia implants positioned in the esthetic jaw areas. The results of this study emphasized that one-piece zirconia dental implants show high biocompatibility, low plaque adhesion, and an absence of a microgap that may contribute to their clinical success in the esthetic areas.

Finally, A. L. Ioannou et al. provide a review of several surgical techniques aimed at optimizing anterior implant esthetics. Clinicians who practice implant dentistry need to strive for more than just osseointegration. They must also achieve excellent esthetic outcomes. Therefore, with careful treatment planning and use of a variety of esthetically focused techniques, clinicians will be able to meet patients' ever-increasing demands for ideal esthetic outcomes.

